# SERS-Based Flavonoid Detection Using Ethylenediamine-β-Cyclodextrin as a Capturing Ligand

**DOI:** 10.3390/nano7010008

**Published:** 2017-01-06

**Authors:** Jae Min Choi, Eunil Hahm, Kyeonghui Park, Daham Jeong, Won-Yeop Rho, Jaehi Kim, Dae Hong Jeong, Yoon-Sik Lee, Sung Ho Jhang, Hyun Jong Chung, Eunae Cho, Jae-Hyuk Yu, Bong-Hyun Jun, Seunho Jung

**Affiliations:** 1 Center for Biotechnology Research in UBITA (CBRU), Institute for Ubiquitous Information Technology and Application (UBITA), Konkuk University, Seoul 05029, Korea; jm.choi.25@gmail.com (J.M.C.); echo@konkuk.ac.kr (E.C.); 2 Department of Bioscience and Biotechnology, Konkuk University, Seoul 05029, Korea; dkhei0525@hanmail.net (E.H.); Rho72@snu.ac.kr (W.-Y.R.); 3 Department of Bioscience and Biotechnology, Microbial Carbohydrate Resource Bank (MCRB) & Center for Biotechnology Research in UBITA (CBRU), Konkuk University, Seoul 05029, Korea; kyeonghee17@naver.com (K.P.); darkamir@nate.com (D.J.); 4 Department of Chemistry Education, Seoul National University, Seoul 08826, Korea; susia45@snu.ac.kr (J.K.); jeongdh@snu.ac.kr (D.H.J.); 5 School of Chemical and Biological Engineering, Seoul National University, Seoul 08826, Korea; yslee@snu.ac.kr; 6 Department of Physics, Konkuk University, Seoul 05029, Korea; shjhang@konkuk.ac.kr (S.H.J.); hjchung@konkuk.ac.kr (H.J.C.); 7 Departments of Bacteriology and Genetics, University of Wisconsin-Madison, Madison, WI 53706, USA; jyu1@wisc.edu

**Keywords:** cyclodextrin, ethylenediamine cyclodextrin, surface-enhanced Raman scattering (SERS), flavonoids

## Abstract

Ethylenediamine-modified β-cyclodextrin (Et-β-CD) was immobilized on aggregated silver nanoparticle (NP)-embedded silica NPs (SiO_2_@Ag@Et-β-CD NPs) for the effective detection of flavonoids. Silica NPs were used as the template for embedding silver NPs to create hot spots and enhance surface-enhanced Raman scattering (SERS) signals. Et-β-CD was immobilized on Ag NPs to capture flavonoids via host-guest inclusion complex formation, as indicated by enhanced ultraviolet absorption spectra. The resulting SiO_2_@Ag@Et-β-CD NPs were used as the SERS substrate for detecting flavonoids, such as hesperetin, naringenin, quercetin, and luteolin. In particular, luteolin was detected more strongly in the linear range 10^−7^ to 10^−3^ M than various organic molecules, namely ethylene glycol, β-estradiol, isopropyl alcohol, naphthalene, and toluene. In addition, the SERS signal for luteolin captured by the SiO_2_@Ag@Et-β-CD NPs remained even after repeated washing. These results indicated that the SiO_2_@Ag@Et-β-CD NPs can be used as a rapid, sensitive, and selective sensor for flavonoids.

## 1. Introduction

Flavonoids are secondary metabolites found in several fruits, grains, flowers, vegetables, teas, and wines [1]. They are important pigments in flowers, and are responsible for ultraviolet (UV) filtration and symbiotic nitrogen fixation [2]. Furthermore, they serve as cell cycle inhibitors or chemical messengers, and have interesting beneficial effects on human health in various applications [3,4,5,6,7,8]. Typically, naturally occurring flavonoids are classified as flavonols, flavonones, flavones, flavanonols, flavans, isoflavonoids, or anthocyanidins. In particular, luteolin (3′,4′,5,7-tetrahydroxyflavone) has been reported to exhibit antimicrobial, anti-inflammatory, and anticancer activities [9,10,11]. Despite these interesting properties, analytical methods for the detection of flavonoids have been limited to chromatography [12,13], capillary electrophoresis [14], and flow injection analysis [15], which are laborious and time-consuming. Moreover, UV detectors show relatively low sensitivity, and capillary electrophoresis is not selective for flavonoids [14].

Surface-enhanced Raman scattering (SERS) is a sensitive analytical technique for in situ detection [16,17] of various molecules such as flavonoids. A majority of studies have focused on the detection of target molecules located near a metal nanoparticle (NP) surface; these molecules are analyzed using their target SERS peaks. However, this direct detection method has several disadvantages, owing to the lack of specificity and different affinities of analytes for the metal surface. Hence, ligands and small organic molecules with high affinities for the target are introduced onto the metal surface in order to detect the target specifically. Although this approach can be used to overcome the limitations associated with the direct detection method, only a few ligands have been reported thus far.

Cyclodextrins (CDs) are cyclic oligosaccharides composed of six, seven, or eight d-glucopyranose units attached through α-1,4 linkages [18]. CDs have a rigid, torus-shaped hydrophobic cavity, which can form inclusion complexes with a wide range of guest molecules [19,20]; this property has been exploited for the detection of flavonoids [21,22,23]. Although various applications of CDs have been reported [24,25,26,27], native CDs still have limitations [28]. To overcome their limited recognition ability, various CD modification methods have been reported [29].

Despite the importance of flavonoids, their selective detection using CD-immobilized nanoparticles has not been reported yet. There are several studies on the identification of flavonoid structures, the identification of the two labile chemical groups of flavonoids [30,31,32,33], and the prediction of the properties of flavones using SERS [33,34,35]. The structural change of quercetin in the presence of metal ions has also been investigated using SERS [36]. SERS has also been used to identify flavonoids that originate from plants and to determine the main efficacious ingredients in the extract of wild *Rubus parvifolius* Linn (RPL) [37]. Given their importance, it is imperative to utilize selective analytical techniques to detect and identify flavonoid molecules in a mixture.

Recently, our group reported the use of β-CD-dimer-assembled SERS substrates, which generate significantly enhanced Raman signals, for the detection of polycyclic aromatic hydrocarbons (PAHs) [38]. In this study, ethylenediamine-modified β-CD (Et-β-CD), which exhibits high affinity for flavonoids, was immobilized on Ag-NP-embedded silica NPs (SiO_2_@Ag@Et-β-CD NPs), which were used as a powerful SERS substrate for the detection of flavonoids, namely hesperetin (Hes), naringenin (Nar), quercetin (Que), and luteolin (Lut). In addition, SiO_2_@Ag@Et-β-CD NPs detected flavonoids selectively, providing a tool for quantitative Lut detection.

## 2. Results and Discussion

### 2.1. Preparation of Et-β-CD-Immobilized Ag-NP-Embedded Silica NPs (SiO_2_@Ag@Et-β-CD NPs)

SiO_2_@Ag@Et-β-CD is composed of Et-β-CD and Ag-NP-assembled nanomaterials. To determine the colloidal stability of SiO_2_@Ag, the zeta potential was measured using a zeta potential analyzer. The zeta potential value of pristine SiO_2_@Ag was −30.88 mV. After conjugation with Et-β-CD, the zeta potential was 41.58 mV. These high zeta potential values of both the SiO_2_@Ag and SiO_2_@Ag@Et-β-CD NPs indicated good colloidal stability. In general, particles in suspension have either a large negative or positive zeta potential value; as a result, there would be no chance of aggregation [39]. Et-β-CD comprises amine groups, which exhibit affinity for silver and serve as ligands for capturing flavonoids via host–guest inclusion complexation (Figure S1). As an SERS substrate, the Ag-NP-embedded silica NPs (Ag NPs@SiO_2_) can enhance SERS signals via their assembled nanostructure [38,40]. For the preparation of the Et-β-CD-immobilized Ag-NP-assembled nanomaterials, Et-β-CD and Ag-NP-assembled nanomaterials were synthesized according to a previously reported procedure [38].

#### 2.1.1. Characterization of Et-β-CD

Et-β-CD was synthesized through monotosylation followed by reaction with ethylenediamine, as described in the experimental section (Figure S1). The structure of synthesized Et-β-CD was confirmed by ^1^H and ^13^C nuclear magnetic resonance (NMR) spectroscopy. In the ^1^H NMR spectrum, the signals observed at 5.05, 3.64, 3.84, 3.57, 3.95, and 3.86 ppm (Figure S2a) corresponded to protons H1 to H6 of the glucopyranose unit, respectively. The peaks of protons H7 and H8 in the ethylenediamine moiety were observed at 2.76 and 2.73 ppm, respectively. In the ^13^C NMR spectra, signals observed at 101.8, 72.1, 73.1, 81.2, 70.5, and 60.3 ppm corresponded to carbons C1–C6, respectively (Figure S2b). In addition, the peaks of carbons C7 and C8, in the ethylenediamine moiety, were observed at 39.4 and 49.0 ppm, respectively. Furthermore, the peak for the substituted C6’ carbons was slightly shifted (49.2 ppm), and distinct from the signal of the unsubstituted C6 carbon (60.3 ppm). NMR spectroscopy data indicated that the ethylenediamine moiety was attached to the C6 atom of native β-CD and that Et-β-CD was synthesized successfully.

#### 2.1.2. UV Absorption Spectra of the Inclusion Complex of Et-β-CD with Flavonoids

**Figure 1:**
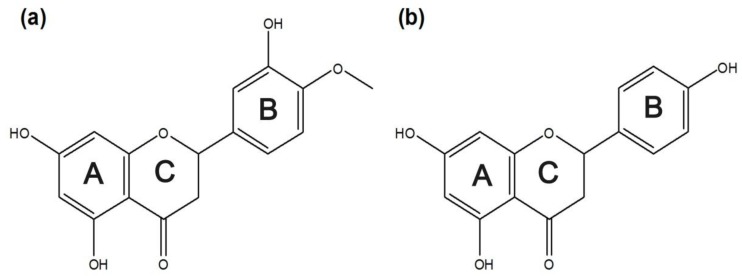

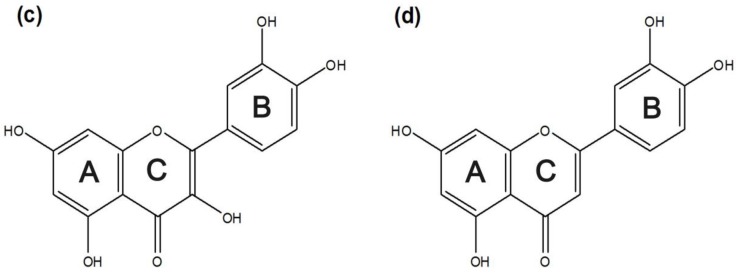
Chemical structures of the flavonoids: (**a**) hesperetin; (**b**) naringenin; (**c**) quercetin; and (**d**) luteolin.

**Figure 2:**
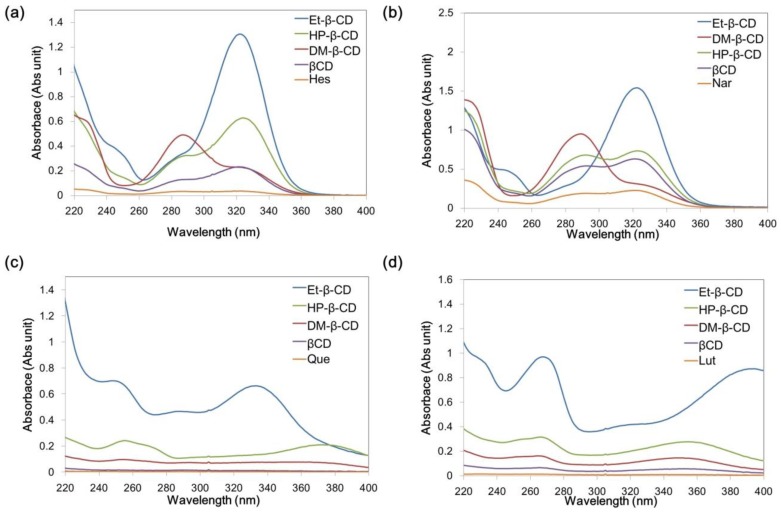
Ultraviolet-visible (UV-Vis) absorption spectra of the flavonoids and their complexes: (**a**) Hesperetin (Hes); (**b**) Naringenin (Nar); (**c**) Quercetin (Que); and (**d**) Luteolin (Lut) in the absence (orange) and presence of β-cyclodextrin (β-CD, purple), dimethyl-β-CD (DM-β-CD, red), 2-hydroxypropyl-β-CD (HP-β-CD, green), and ethylenediamine-modified β-CD (Et-β-CD, blue).

Figure 1 shows the chemical structures of the four flavonoids. Figure 2 shows their UV absorption spectra in the absence and presence of 2 mM β-CD, 2-hydroxypropyl-β-CD (HP-β-CD), and Et-β-CD. Figure 2a,b shows the UV absorption spectra of Hes and Nar with various β-CD derivatives. The λ_max_ of Hes (or Nar) and its corresponding complexes with β-CD, HP-β-CD, and Et-β-CD was at 322 nm, and the λ_max_ of the Hes (or Nar) complex with dimethyl-β-CD (DM-β-CD) was at 288 nm. These blue-shifts in the spectra were attributed to hydrogen bonds between the host DM-β-CD and Hes (or Nar), possibly because hydrogen bonding can lower the energy of the “n” (non-bonding) orbitals [41]. Hydrogen bonding is considered one of the main forces driving the formation of an inclusion complex. UV absorption was primarily enhanced upon complexation of Et-β-CD with Hes (or Nar). Figure 2c,d shows the UV absorption spectra of the Que and Lut complexes, respectively; their UV absorptions also increased because of complexation. Enhancement of UV absorption followed the order of Et-β-CD > HP-β-CD > DM-β-CD > β-CD. Enhanced UV absorption was observed for Et-β-CD with all four flavonoids. The enhancement was caused by the outstanding complex-forming ability of Et-β-CD compared to those of the other β-CD derivatives. During complexation, guest molecules were transferred from an aqueous medium to the hydrophobic environment, resulting in UV absorption changes [42,43,44,45,46]. In addition, significant differences in UV absorption were not observed for the different flavonoids (Figure S3). The maximum absorbance peaks for SiO_2_@Ag@Et-β-CD, after reacting with Lut or Que, increased in intensity, owing to the combination effect on the extinction coefficient of the nanomaterials due to visible light. In contrast, marginal changes in absorbance intensity were observed with other flavonoids, namely Nar and Hes.

#### 2.1.3. Preparation of SiO_2_@Ag@Et-β-CD

Ag-NP-assembled silica NPs were prepared according to a previously reported method [38,40]. Silica NPs, as the core, were synthesized using the Stöber method [47], and their size was controlled to be approximately 180 nm. Hydroxyl groups present on the silica NP surface were converted to thiols, and the Ag NPs were introduced directly onto the modified surface via the reduction of AgNO_3_ in solution. Figure S4 shows the transmission electron microscopy (TEM) image of the prepared SiO_2_@Ag NPs. As amine groups exhibit affinity for metals and form amine-metal complexes, Et-β-CD can be immobilized on the SiO_2_@Ag NPs by simple mixing with stirring. To examine the optical properties of SiO_2_@Ag, UV-visible (UV-Vis) spectra were recorded (Figure S2). The UV-Vis spectra of both unmodified and modified SiO_2_@Ag with Et-β-CD displayed a broad band from 325 to 650 nm. In particular, in both cases, a maximum peak at around 430 nm was observed, corresponding to Ag NPs on the silica NP surface. After the functionalization of Et-β-CD on SiO_2_@Ag, the maximum peak decreased in intensity and shifted slightly from 425 to 435 nm.

### 2.2. Detection of Flavonoids via SERS Using SiO_2_@Ag@Et-β-CD NPs

To verify whether SiO_2_@Ag@Et-β-CD NPs can detect flavonoids effectively and to evaluate the detection ability and stability of SiO_2_@Ag@Et-β-CD NPs as a sensor, systematic studies were performed with various flavonoids.

#### 2.2.1. Detection of Various Flavonoids Using Et-β-CD-Immobilized Ag-NP-Assembled Silica NPs

**Figure 3:**
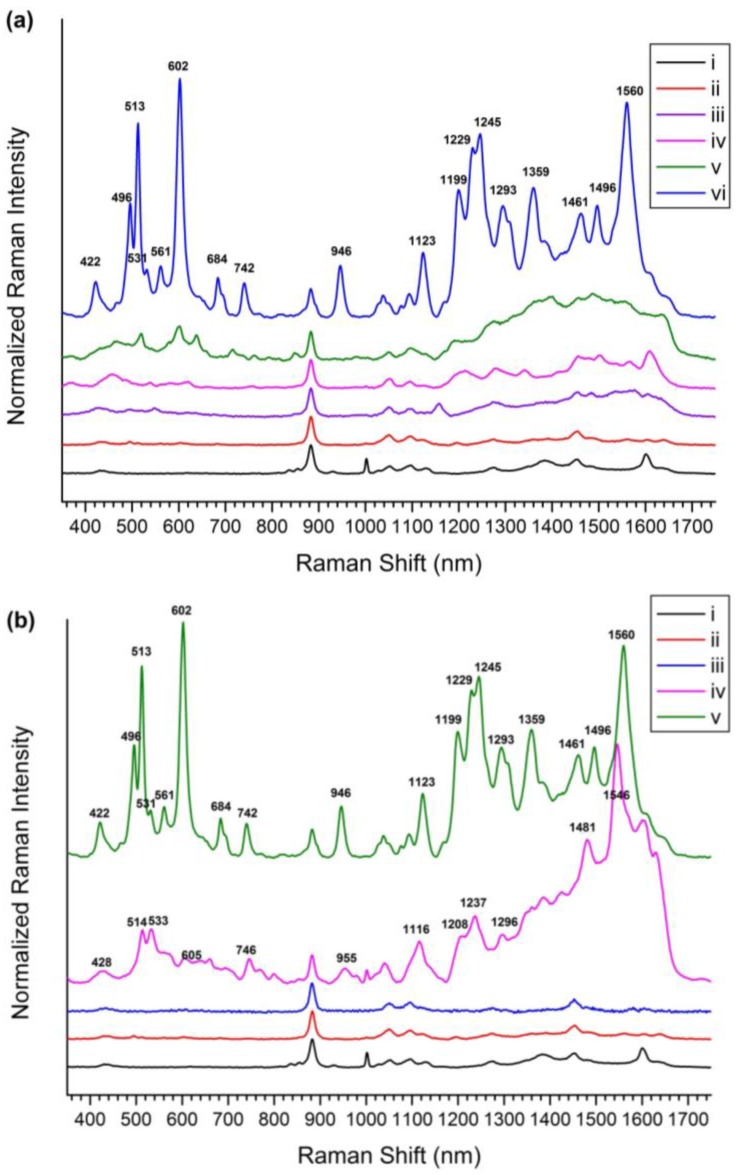
Surface enhanced Raman scattering (SERS) spectra. (**a**) SERS spectra of (i) silver nanoparticle (NP)-embedded silica NPs (SiO_2_@Ag NPs), (ii) SiO_2_@Ag@Et-β-CD NPs, (iii) SiO_2_@Ag@Et-β-CD NPs with Nar, (iv) SiO_2_@Ag@Et-β-CD NPs with Hes, (v) SiO_2_@Ag@Et-β-CD NPs with Que, and (vi) SiO_2_@Ag@Et-β-CD NPs with Lut; (**b**) SERS spectra of (i) SiO_2_@Ag NPs, (ii) SiO_2_@Ag@Et-β-CD NPs, (iii) Lut in ethanol (10^−2^ M), (iv) SiO_2_@Ag NPs mixing with Lut (10^−4^ M), and (v) SiO_2_@Ag@Et-β-CD NPs mixing with Lut (10^−4^ M).

For flavonoid detection, a SiO_2_@Ag@Et-β-CD NP solution was mixed with the target molecules for 1 h and transferred to capillary tubes, and then Raman spectra were measured. To investigate the flavonoid detection capability of the SiO_2_@Ag@Et-β-CD NPs, they were mixed with Nar, Hes, Que, and Lut (10^−4^ M), and the SERS spectra were recorded (Figure 3a). In the absence of flavonoids, as the control studies, the spectrum of the SiO_2_@Ag NPs in ethanol (EtOH) showed only the peaks of ethanol and polyvinylpyrrolidone (PVP). The prominent band at 883 cm^−1^ belonged to EtOH, and other smaller bands at 1001, 1053, 1095, and 1600 cm^−1^ characteristic of PVP also appeared. When Et-β-CD was attached to the SiO_2_@Ag NPs as a linker without any flavonoids, the resulting SiO_2_@Ag@Et-β-CD NPs in EtOH exhibited a prominent EtOH band at 883 cm^−1^ with other smaller bands at 1050, 1095, and 1455 cm^−1^. However, the bands at 1001 and 1600 cm^−1^ were absent, which indicated that PVP could be removed from the nanoparticle surface via replacement with a reactive molecule. However, in the presence of flavonoids, the SERS spectra of the SiO_2_@Ag@Et-β-CD NPs showed different characteristic SERS bands depending on the binding flavonoid. For example, the spectrum of Nar showed SERS bands at 548 and 1158 cm^−1^; that of Hes showed SERS bands at 457, 620, 1278, and 1342 cm^−1^; and that of Que showed SERS bands at 520, 715, and 848 cm^−1^. The spectrum of Lut with the SiO_2_@Ag@Et-β-CD NPs showed significantly stronger SERS bands at 422, 496, 513, 561, 684, 742, and 1123 cm^−1^. These results show that SiO_2_@Ag@Et-β-CD NPs exhibit high affinity for Lut; this selectivity was caused by the structural differences of the four flavonoids (Figure 1). The characteristics of the guest molecules, such as structure-dependent properties like hydrophobicity and polarizability, may affect the selectivity of the complex. The spectrum of Lut showed the highest SERS intensity after binding with CD because of the differences in the electrostatic interactions of each complex, particularly hydrogen bonding [20,48]. To investigate the effect of a linker, we measured the SERS signals of model flavonoid Lut in the presence of SiO_2_@Ag NPs with and without Et-β-CD (Figure 3b). Lut in EtOH did not show its characteristic peaks except for the EtOH peaks, even though a high concentration was introduced (Figure 3b(iii)), owing to its low scattering extinction. However, Lut with and without Et-β-CD showed its characteristic peaks. In the absence of Et-β-CD (Si@Ag), Lut showed characteristic peaks at 428, 514, 533, 605, 746, 955, 1116, 1208, 1237, 1296, 1481, and 1546 cm^−1^ because of the electromagnetic enhancement caused by the SiO_2_@Ag NPs (Figure 3b(vi)). In contrast, in the presence of SiO_2_@Ag@Et-β-CD NPs, the characteristic peaks of Lut were observed at 422, 496, 513, 531, 561, 602, 684, 742, 946, 1123, 1199, 1229, 1245, 1293, 1359, 1461, 1496, and 1560 cm^−1^ (Figure 3b(v)). Comparison of these two spectra showed that the peaks of Lut with SiO_2_@Ag@Et-β-CD NPs were slightly shifted. The appearance of the new peaks at 496, 561, 684, 1245, 1359, 1461, and 1496 cm^−1^ and the increases in peak intensity at 513, 602, 742, 946, 1199, 1229, and 1293 cm^−1^ confirmed that Lut interacted selectively with Et-β-CD, causing the functional group of Lut to move near the surface of the Ag NPs.

Subsequently, additional experiments were conducted to evaluate the selectivity of the SiO_2_@Ag@Et-β-CD NPs for Lut. SiO_2_@Ag@Et-β-CD NPs were mixed with different organic compounds (ethylene glycol, β-estradiol, isopropyl alcohol, naphthalene, toluene, or Lut) at a concentration of 10^−4^ M. Unlike the mixtures containing flavonoids and the SiO_2_@Ag@Et-β-CD NPs, the organic-compound-containing solutions did not show any characteristic peaks in their Raman spectra (Figure S5a). To further study the selectivity of the SiO_2_@Ag@Et-β-CD NPs, another control experiment involving aniline with SiO_2_@Ag NPs and SiO_2_@Ag@Et-β-CD NPs was conducted, and the results are displayed in Figure S4b. Aniline can bind to a metal through the non-paired electrons of its amino group. Its characteristic SERS peaks were observed at 1236, 1550, and 1581 cm^−1^ upon binding with SiO_2_@Ag NPs (Figure S5b(ii)). However, these characteristic peaks disappeared upon binding with SiO_2_@Ag@Et-β-CD NPs (Figure S5b(iii)). We believe that the Et-β-CD on the surface of the SiO_2_@Ag NPs hinder the attachment of aniline to their surface. In the SERS spectrum of the mixed solution of aniline and Lut with SiO_2_@Ag@Et-β-CD NPs, only the characteristic peaks of Lut at 604, 942, 1203, 1226, and 1558 cm^−1^ are shown (Figure S5b(iv)). These results indicate that the SiO_2_@Ag@Et-β-CD NPs can be used for detecting flavonoids selectively. Therefore, SiO_2_@Ag@Et-β-CD exhibited selectivity towards Lut, owing to the recognition function of Et-β-CD, as can be seen by comparing Figure 3a line (vi) with the SERS spectra of complexes formed via non-specific interactions between the other flavonoids and pristine SiO_2_@Ag shown in Figure S6.

#### 2.2.2. Determination of the Detection Limit of SiO_2_@Ag@Et-β-CD NPs for Lut

**Figure 4:**
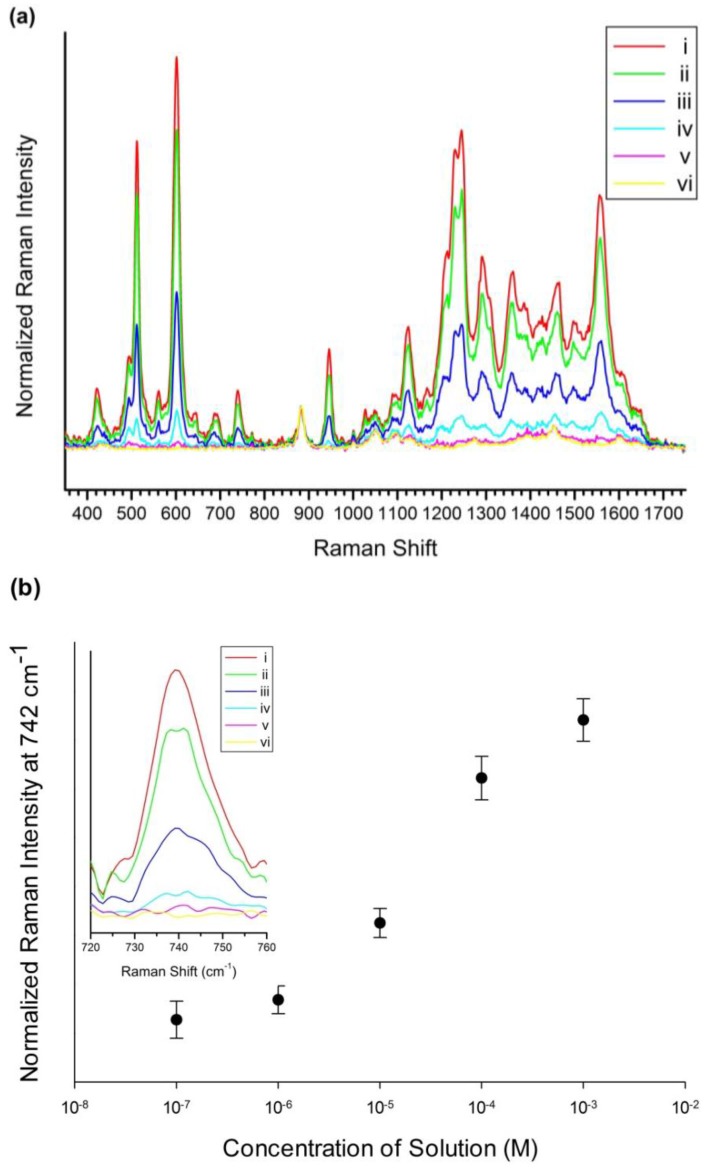
SERS spectra and normalized SERS intensity. (**a**) SERS spectra of SiO_2_@Ag@Et-β-CD NPs mixed with Lut at concentrations from 1 × 10^−3^ M to 1 × 10^−7^ M; (**b**) Normalized SERS intensities at 742 cm^−1^ ((i) 10^−3^ M, (ii) 10^−4^ M, (iii) 10^−5^ M, (iv) 10^−6^ M, (v) 10^−7^ M, and (vi) 0 M).

To investigate the capability of the SiO_2_@Ag@Et-β-CD NPs for quantitative flavonoid detection, a SiO_2_@Ag@Et-β-CD NP solution was treated with Lut solutions of various concentrations from 1 × 10^−3^ M to 1 × 10^−7^ M for 1 h. Figure 4a shows the Raman spectra of the SiO_2_@Ag@Et-β-CD NPs mixed with Lut. The SERS intensity increased with the Lut concentration in the studied range, particularly the intensities of the peaks observed at 513, 684, 742, and 1123 cm^−1^. Figure 4b shows the normalized SERS intensity at 742 cm^−1^. The detection limit for Lut based on the SERS signals of the SiO_2_@Ag@Et-β-CD NPs was 10^−7^ M.

#### 2.2.3. Stability of the Flavonoid Captured by Et-β-CD-Immobilized Ag-NP-Assembled Silica NPs

**Figure 5:**
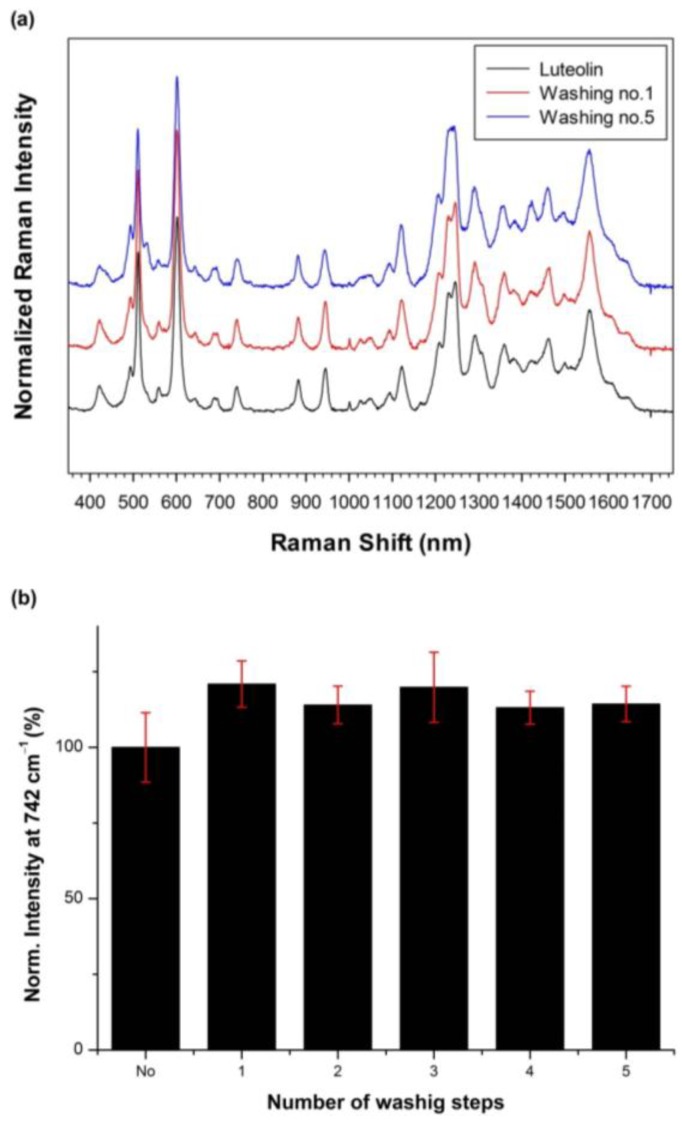
(**a**) Raman spectra and (**b**) normalized intensities of Lut captured by SiO_2_@Ag@Et-β-CD NPs at 742 cm^−1^ after washing with ethanol (Lut concentration, 10^−4^ M).

To investigate the stability of Lut captured by SiO_2_@Ag@Et-β-CD NPs, the SiO_2_@Ag@Et-β-CD NPs were washed several times with ethanol to dissolve Lut, and the Raman intensity of the captured Lut at 742 cm^−1^ was measured after each washing. Figure 5a shows the Raman spectra of Lut captured by SiO_2_@Ag@Et-β-CD NPs after each wash with ethanol, and Figure 5b shows the normalized Raman intensities at 742 cm^−1^. No significant changes in the Raman intensity were observed after washing. Therefore, Lut captured by the SiO_2_@Ag@Et-β-CD NPs is stable even after washing five times.

## 3. Materials and Methods

### 3.1. Chemicals

β-CD (>95.0%, high-performance liquid chromatography, M_w_ = 1134.99 Da), 1-(***p***-toluenesulfonyl)imidazole, Lut, Nar, and ethylenediamine were obtained from Tokyo Chemical Industry Co., Ltd. (Tokyo, Japan) HP-β-CD (M_n_ = 1460 Da, 0.8 molar substitution), Hes, Que dehydrate, and DM-β-CD were purchased from Sigma Aldrich (St. Louis, MO, USA). Deionized water was obtained from a Milli-Q system (Millipore, Saint-Quentin-en-Yvelines, France). Analytical-grade chemicals were used as received.

### 3.2. Synthesis of Ethylenediamino-β-Cyclodextrin (Et-β-CD)

First, tosylated β-CD (3 g, 2.33 mmol) (Tos-β-CD, supplied by the Microbial Carbohydrate Resource Bank of Konkuk University, Seoul, Korea) was dissolved in ethylenediamine (10 mL) under N_2_ [49]. Then, the mixture was stirred at 75 °C for 12 h. After the reaction mixture had cooled to room temperature, a large volume of acetone (three volumes) was added for precipitation. The precipitated samples were dissolved in distilled water and separated using CM Sephadex C-25. After column separation, the samples were desalted using Bio-Gel P-2. The purity of the obtained solid (1 g, 0.85 mmol) was confirmed by matrix-assisted laser desorption/ionization time-of-flight (MALDI-TOF) mass spectrometry (Voyager-DE™ STR Bio-Spectrometry, Applied Biosystems, Framingham, MA, USA) and 600 MHz NMR spectroscopy (Bruker Avance 600 spectrometer, Billerica, MA, USA) [50].

### 3.3. UV Absorption Measurement of the Inclusion Complex

First, an excess of each flavonoid (500 μM) was added to 2 mM of each of the aqueous solutions of β-CD, HP-β-CD, DM-β-CD, and Et-β-CD in capped vials, and each of the solutions was subjected to sonication for 10 min. Then, the solutions in the vials were stirred for 24 h at 25 °C and kept in the dark to prevent degradation. After equilibrium was attained, the complexes were filtered using a 0.2 μm polyvinylidene fluoride filter. Each sample was analyzed using a UV-Vis spectrophotometer (UV 2450, Shimadzu Corporation, Kyoto, Japan) from 220 to 400 nm.

### 3.4. Preparation of SiO_2_@Ag@Et-β-CD NPs

Ag-NP-assembled silica NPs (SiO_2_@Ag) were synthesized according to previously reported methods [38]. The mass of the SiO_2_@Ag NPs was determined as the dry weight at a specific volume. SiO_2_@Ag NPs (8 mg) were mixed with an Et-β-CD solution (4 mM in water). The resulting suspension was stirred vigorously for 12 h at 25 °C, centrifuged (13,000 rpm, 10 min), washed with EtOH, and re-dispersed in absolute EtOH (0.8 mL). To determine the zeta potential of SiO_2_@Ag, its colloidal stability was determined using a zeta potential analyzer (ELS-8000, Otsuka Electronics, Osaka, Japan).

### 3.5. Interactions of SiO_2_@Ag@Et-β-CD NPs with Flavonoids and Organic Molecules

Each flavonoid and organic molecule stock solution (100 mM) was prepared using absolute ethanol. The prepared SiO_2_@Ag@Et-β-CD NPs (0.5 mg in 50 μL of EtOH) were mixed with the stock solutions after diluting to the desired concentrations and then the mixtures were shaken for 1 h under ambient conditions.

### 3.6. Raman Spectral Measurements

To evaluate the sensitivity of the synthesized SERS materials, they were transferred to a capillary tube and measured using a DXR™ Raman Microscope system (Thermo Fisher Scientific, Waltham, MA, USA). The SERS spectra were recorded in backscattering geometry using a 10× objective lens. A 532 nm diode-pumped solid-state laser was used as the photoexcitation source, with a laser power of 5 mW. Selected sites were randomly measured, and all SERS spectra were integrated for 2 s. The spot size of the laser beam was ~2 μm.

## 4. Conclusions

In this study, Et-β-CD-immobilized Ag-NP-embedded silica NPs (SiO_2_@Ag@Et-β-CD NPs) were prepared for the detection of flavonoids (i.e., Hes, Nar, Que, and Lut). Based on its high affinity for flavonoids, Et-β-CD could be used as a ligand for their SERS detection. As the strongest SERS bands were observed for Lut, the selectivity and sensitivity in the detection of Lut using SiO_2_@Ag@Et-β-CD NPs were also investigated. In this system, the limit of detection (LOD) for Lut was 10^−7^ M, and the Lut captured by SiO_2_@Ag@Et-β-CD NPs was stable even after washing five times. Based on this study, we propose the use of SiO_2_@Ag@Et-β-CD NPs for the SERS-based detection of flavonoids. In addition, this assembled structure hybridized with capturing ligands serves as a promising platform for the design of sensitive and selective sensing nanomaterials.

## Supplementary Materials

The following are available online at http://www.mdpi.com/2079-4991/7/1/8/s1.
