# Supplementary material for: SERS-Based Flavonoid Detection Using Ethylenediamine-β-Cyclodextrin as a Capturing Ligand
Source: Nanomaterials (Basel). 2017 Jan 6;7(1):8. doi: 10.3390/nano7010008 (PMC5295198; doi:10.3390/nano7010008)
Supplement: Supplementary file 1 [file nanomaterials-07-00008-s001.pdf]

Supplementary Materials: SERS-Based Flavonoid Detection Using Ethylenediamine-β-Cyclodextrin as a Capturing Ligand

Jae Min Choi, Eunil Hahm, Kyeonghui Park, Daham Jeong, Won-Yeop Rho, Jaehi Kim, Dae Hong Jeong, Yoon-Sik Lee, Sung Ho Jhang, Hyun Jong Chung, Eunae Cho, Jae-Hyuk Yu, Bong-Hyun Jun, and Seunho Jung

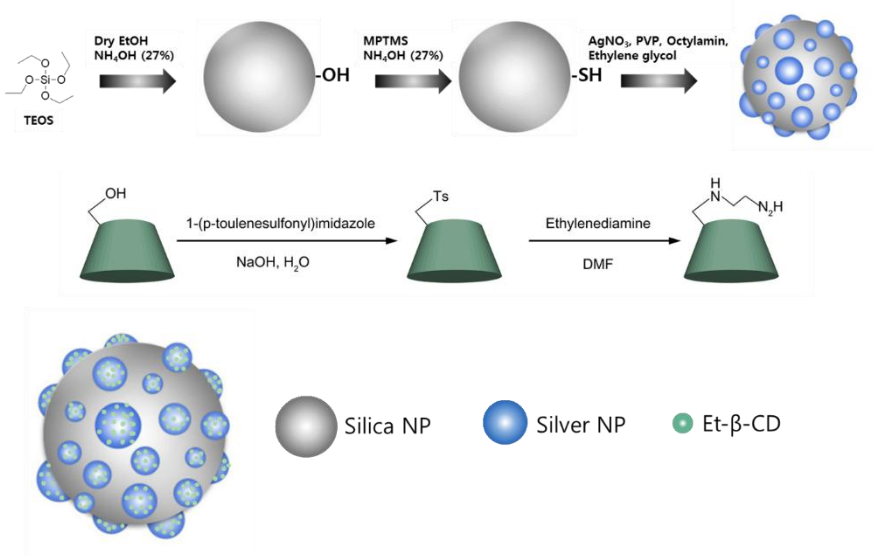

Figure S1. Schematic illustration of the synthesis of Et-β-CD@Ag@SiO2 NPs.

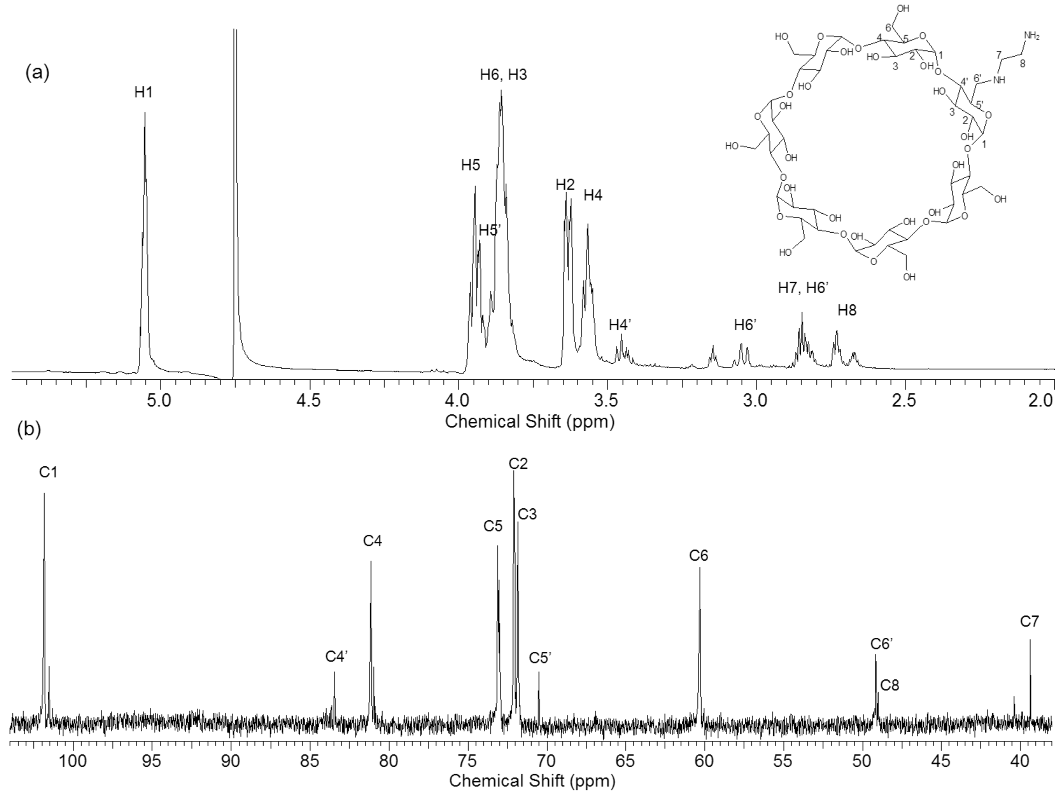

Figure S2. 1D NMR spectra of Et-β-CD. (**a**) ^{1}H NMR spectrum; (**b**) ^{13}C NMR spectrum. The NMR experiments were carried out on a Brucker Avance spectrometer at 600 MHz in a D_2O solution at 25 °C.

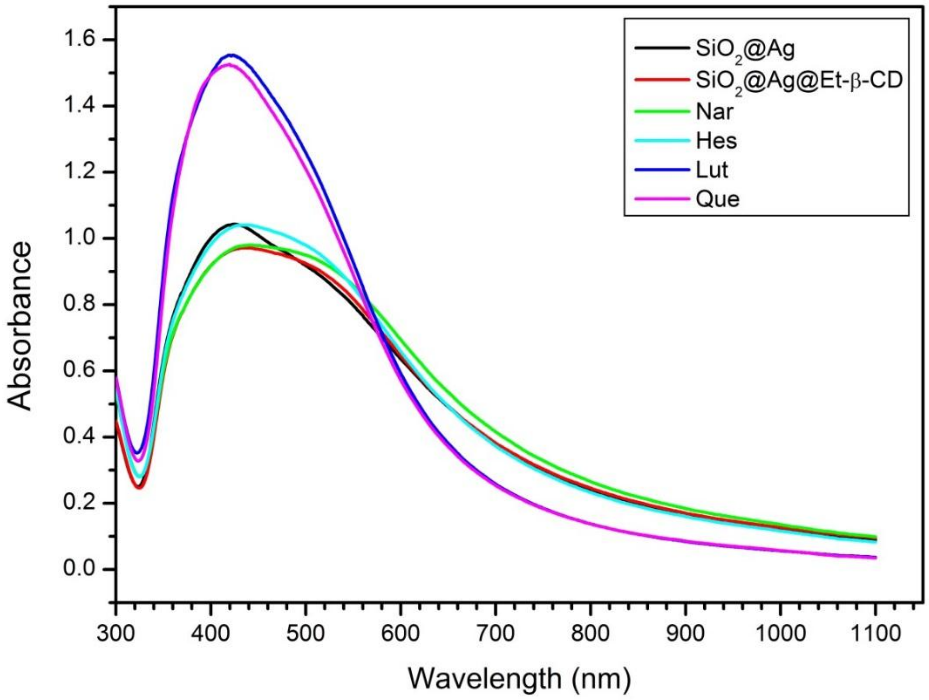

Figure S3. UV-Vis absorbance of SiO_2@Ag@Et-β-CD NPs reacted with Nar, Hes, Lut, and Que.

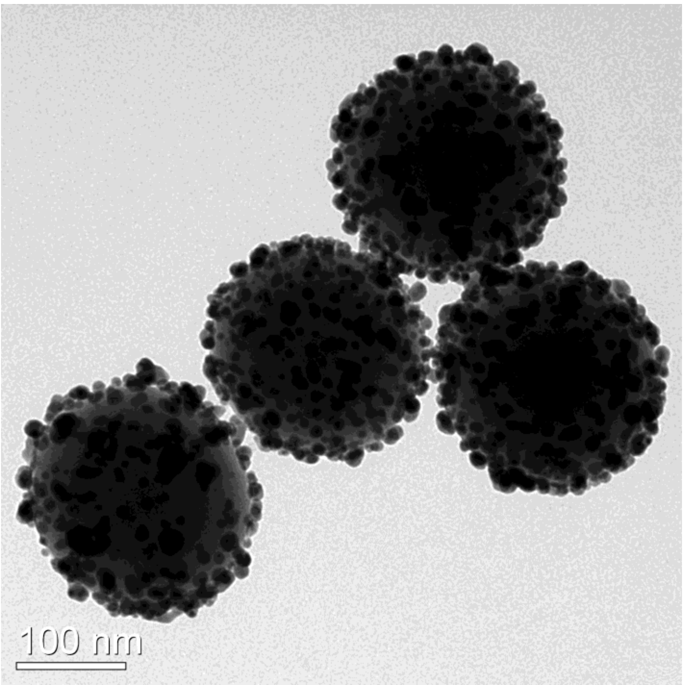

Figure S4. TEM images of Ag@SiO_2 NPs in EtOH Ag@SiO_2 NPs measured using an energy-filtering transmission electron microscope (EF-TEM, LIBRA 120, Carl Zeiss, Oberkochen, Germany). The accelerating voltage was 120 kV.

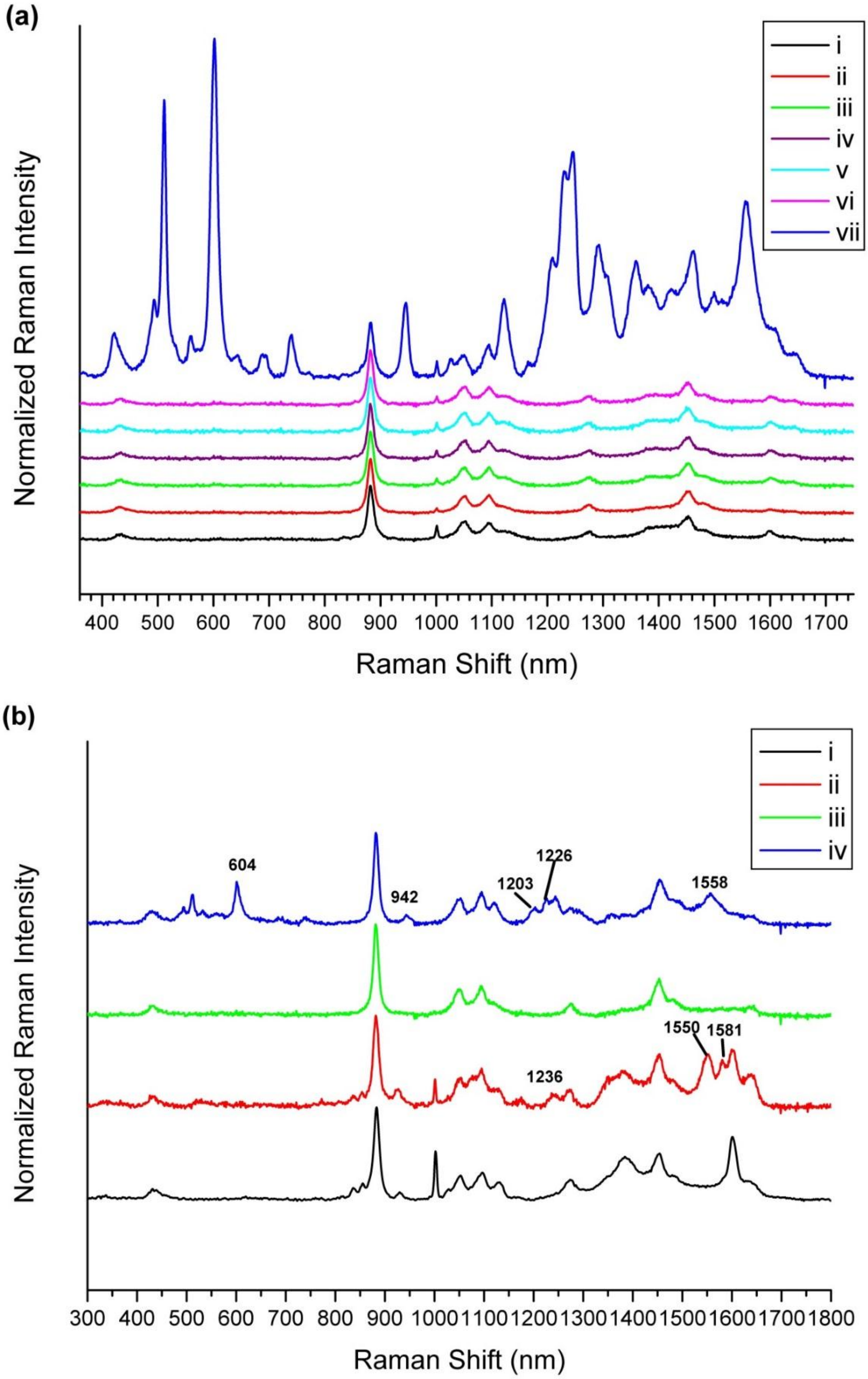

Figure S5. Surface-enhanced Raman scattering (SERS) spectra. (**a**) SERS spectra of organic molecu-les after mixing with Et-β-CD@Ag@SiO_2 NPs (i. no target, ii. ethylene glycol, iii. β-estradiol, iv. Isop-ropyl alcohol, v. naphthalene, vi. toluene and vii. Lut); (**b**) SERS spectra of (i) SiO_2@Ag NPs, (ii) SiO_2@Ag NPs reacted with Aniline, (iii) SiO_2@Ag@Et-β-CD reacted with Aniline, and (iv) SiO_2@Ag@Et-β--CD NPs reacted with mixing solution of Lut and aniline.

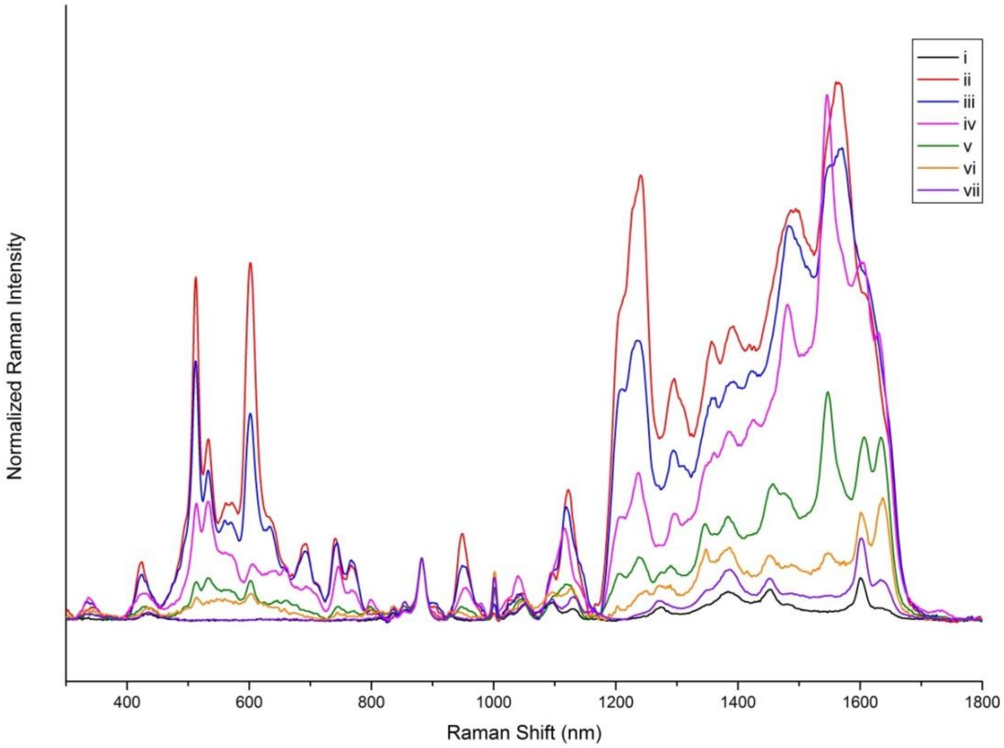

Figure S6. Surface-enhanced Raman scattering (SERS) spectra and normalized SERS intensity graph. SERS spectra of SiO_2@ Ag NPs mixed with Lut at 1 × 10^{-2} M to 1 × 10^{-7} M.
